# Supplementary material for: An Instrument to Measure Mental Health Professionals’ Beliefs and Attitudes towards Service Users’ Rights
Source: Int J Environ Res Public Health. 2019 Jan 16;16(2):244. doi: 10.3390/ijerph16020244 (PMC6352182; doi:10.3390/ijerph16020244)
Supplement: Supplementary file 1 [file ijerph-16-00244-s001.pdf]

International Journal of
Environmental Research and Public Health

MDPI

Table S1. Descriptive data of the initial 44 item pool.

Item	Title	Frequencies (%)				Asymmetry	Kurtosis
		Totally agree	Agree	Disagree	Totally disagree		
1	The possibility of people with severe mental disorders having children should be regulated.	36.4	34.7	23.2	5.7	0.496	−0.733
* 2	*Even in the most serious episodes patients must be involved in the decisions.*	1.5	5.9	42.0	50.6	1.022	1.046
3	*It is possible to recover without professional interventions.*	5.3	31.7	44.5	18.5	0.136	−0.554
4	*People should not be involuntarily hospitalised if they do not pose a threat to the integrity of others*	4.8	37.9	31.2	26.1	−0.017	−1.056
* 5	*Coordination with community leisure and citizen participation services should be a fundamental part of treatment.*	1.0	1.0	20.3	77.6	2.397	7.158
6	Sometimes it is necessary to mechanically restrain patients.	19.5	27.5	44.4	8.6	−0.217	−0.858
* 7	*The patients' perspective should be considered in the understanding of mental disorders.*	0.2	0.8	17.8	81.1	02.166	5.098
8	Patients with severe mental disorders require clearer instructions than other patients..	12.5	39.7	37.4	10.4	0.030	−0.579
9	A hospital admission is a sign of failure for the professional that takes charge of the case.	33.3	50.7	11.3	4.6	0.805	0.516
10	Mental disorders are diseases like any other.	13.7	31.7	32.1	22.5	−0.096	−1.008
11	Professionals should have more say than patients in making treatment decisions.	40.0	47.8	9.9	2.3	0.797	0.516
12	*Individuals incapacitated by severe mental health problems should have the right to vote.*	2.5	12.4	39.3	45.8	0.865	0.171
13	When patients behave aggressively it is due to their mental disorder	32.1	55.0	10.9	1.9	0.591	0.463
14	Declaring someone with a severe mental disorder incapacitated is a good way of taking care of that person.	31.6	48.5	18.6	1.3	0.360	−0.509
15	Individuals with mental disorders currently have the same rights as other people.	30.2	42.8	13.2	13.8	0.648	−0.564
16	Coercive measures are currently applied only when necessary.	22.9	54.1	20.6	2.3	0.328	−0.143
** 17	*A person can recover even if they still display symptoms.*	0.4	1.9	49.3	48.4	0.521	0.129
** 18	The obtainment of a mental health professional qualification should be restricted for individuals with psychiatric records.	49.1	45.1	4.4	1.5	0.991	1.286
** 19	To prevent the misuse of services, consultations of patients with psychiatric records in other specialties should be controlled.	51.1	40.0	8.1	0.8	0.889	0.314
* 20	Most individuals with mental health problems have a lower intellectual capacity than the general population.	66.9	28.3	2.9	1.9	1.794	3.612
21	The family relationships of individuals with mental disorders always have an underlying pathology.	27.5	48.2	21.4	2.9	0.378	−0.377
* 22	*Contact with recovered patients should be a fundamental part of mental health training.*	1.3	1.9	37.3	59.5	1.335	2.429

23	*When a patient behaves aggressively it is due to the situations that occur, for example in involuntary admissions.*	11.9	47.0	33.5	7.5	−0.175	−0.366
24	*I would feel comfortable making friends with someone with a severe mental disorder.*	2.5	10.8	52.9	33.8	0.695	0.541
25	I am uncomfortable with patients who regularly use emergency services.	39.0	48.1	12.0	0.8	0.538	−0.225
* 26	If a patient questions the treatment this is a sign that the disorder is of a greater severity.	55.6	39.4	3.1	1.9	1.311	2.191
27	People with severe mental disorders always require support to be able to live independently.	34.2	47.2	15.7	2.9	0.601	−0.072
28	Objective tests should be prioritised over the professionals' and patients' opinion.	16.1	32.0	43.0	8.9	−0.172	−0.708
* 29	*Support from other people with mental disorders should be integrated into the mental health care system.*	1.5	4.6	44.7	49.2	0.993	1.246
30	*I would be comfortable if a person with a mental disorder were a teacher in a school.*	2.1	12.3	52.4	33.2	0.606	0.283
** 31	Patients with mental disorders unjustifiably visit other health services.	41.9	51.4	6.3	0.4	0.491	−0.057
** 32	*Social inequalities are as important as psychological and biological factors in the genesis of mental disorders.*	2.7	8.4	43.9	45.0	0.975	0.818
33	Respecting the patients' dignity is important, but some aspects of treatment may require flexibility.	29.1	33.9	30.9	6.1	0.209	−0.951
34	*Greater importance should be placed on promoting the patient's independence than on reducing the patient's symptoms.*	0.8	11.2	53.1	34.9	0.458	−0.056
** 35	At a glance I can distinguish a person with mental health problems.	40.0	53.7	5.2	1.0	0.629	0.732
* 36	A hospital admission is a sign of a patient's failure.	57.8	39.5	1.9	0.8	1.104	1.599
37	If there are not enough staff, mechanical restraints are the only way to manage violent situations.	37.4	48.1	12.8	1.7	0.613	0.011
38	When dealing with patients it is important for me not to get emotionally involved.	32.4	45.4	19.7	2.5	0.448	−0.441
39	Some patients will never be able to recover.	29.7	41.7	26.1	2.5	0.257	−0.799
40	In my clinical practice I try to leave my personal values aside.	27.2	41.1	25.9	5.7	0.332	−0.661
** 41	The length of the driving license should be restricted to patients with a history of psychiatric disorders, even if they do not take medication.	45.2	45.2	8.8	0.8	0.713	0.097
** 42	The priority of the treatment should be to alleviate the symptomatology rather than adhere to the patient's preferences.	38.6	52.8	7.3	1.3	0.647	0.578
** 43	*Collecting the preferences of patients in stable phase with respect to their treatment in acute phase would avoid coercive measures.*	0.4	3.2	44.0	52.4	0.746	0.298
44	For the most part, mental health professionals work collaboratively with patients.	9.2	40.0	43.2	7.5	−0.081	−0.366

Italicized items are considered reverse. * Removed due to asymmetry and kurtosis scores. ** Removed due to low discriminative capacity (90% of the cases included in one of the two halves of the Likert scale).

Table S2. Evolution of confirmatory factor analysis fit and discrimination parameters.

	MFTS	CFI	TLI	RMSEA	SRMR	IRT/MIRT
**CFAs analysing the unidimensionality of the whole structure**						**New item discrimination *****
Unidimensional core structure	479 *	0.798	0.772	0.069	0.063	
Unidimensional core structure + item 11	507 *	0.809	0.787	0.066	0.061	1.843
Unidimensional core structure + item 13	505 *	0.800	0.777	0.066	0.061	0.824
Unidimensional core structure + item 14	516 *	0.809	0.787	0.067	0.061	1.765
*Unidimensional core structure + item 21 ***	*539 **	*0.779*	*0.753*	*0.069*	*0.065*	*0.413*
Unidimensional core structure + item 33	512 *	0.799	0.775	0.067	0.061	1.006
Unidimensional core structure + item 37	522 *	0.794	0.770	0.067	0.062	0.960
Unidimensional core structure + item 39	513 *	0.804	0.781	0.067	0.062	1.266
Unidimensional final structure (25 items)	704 *	0.821	0.805	0.059	0.057	
**CFAs considering each substructure separately**						New item discrimination ****
Core structure (19 items)	298 *	0.906	0.890	0.048	0.051	Unidimensional/ Multidimensional
Core structure + item 11 (Paternalism)	341 *	0.900	0.884	0.049	0.052	1.402/1.164
Core structure + item 13 (Justifying beliefs)	324 *	0.905	0.889	0.046	0.051	0.735/0.723
Core structure + item 14 (Justifying beliefs)	362 *	0.891	0.873	0.052	0.052	1.223/1.205
Core structure + item 21 (Discrimination)	*351 **	*0.888*	*0.870*	*0.050*	*0.055*	*0.643/0.625*
Core structure + item 33 (Paternalism)	336 *	0.899	0.883	0.048	0.051	0.907/0.914
Core structure + item 37 (Coercion)	342 *	0.896	0.879	0.049	0.052	0.898/0.932
Core structure + item 39 (Justifying beliefs)	363 *	0.886	0.868	0.052	0.054	0.758/0.744
**Final structure (25 items)**	**590 ***	**0.866**	**0.851**	**0.051**	**.053**	
**Unidimensionality of final subscales**						
Justifying beliefs (items 3, 10, 13, 14, 15, 16, 39 and 44)	70 *	0.910	0.874	0.073	0.048	
Coercion (items 4, 6, 23, 34, and 37)	10	0.980	0.959	0.049	0.029	
Paternalism (items 1, 8, 11, 27, 28, 33, 38, and 40)	61 *	0.914	0.880	0.067	0.046	
Discrimination (items 12, 24, 25, and 30)	2	1	0.999	0.011	0.014	

MFTS: Minimum Function Test Statistic, CFI: Comparative Fix Index, TLI: Tucker Lewis Index, RMSEA: Root Mean Square Error of Approximation, SRMR: Standardized Root Mean Square Residual. IRT: Item Response Theory, MIRT: Multidimensional Item Response Theory. Core structure: justifying beliefs (items 3, 10, 15, 16, 44), coercion (items 4, 6, 23, 34), paternalism (items 1, 8, 27, 28, 38, 40) and discrimination (items 12, 24, 25, 30). *p<.0001. **Item 21 was excluded as it does not improve fit and discrimination parameters are generally low. Discrimination for items added using unidimensional IRT unconstrained latent variable models within the whole model (***) and within each substructure unidimensional or using confirmatory MIRT (****).
